# Use of Web-Based Patient Portals in Patients With Atrial Fibrillation Is Associated With Higher Readmissions

**DOI:** 10.31486/toj.19.0124

**Published:** 2021

**Authors:** Arthur P. Davis, Gibbs M. Wilson, John P. Erwin, Jeffrey B. Michel, Javier Banchs, Aasim Saeed, Robert J. Widmer

**Affiliations:** Department of Internal Medicine, Baylor Scott and White Medical Center, Temple, TX

**Keywords:** *Atrial fibrillation*, *patient portals*, *patient readmission*

## Abstract

**Background:** The impact of web-based patient portals on patient outcomes—specifically hospital readmissions in patients with atrial fibrillation (AF)—remains understudied.

**Methods:** This single-center retrospective cohort study investigated the use of an online portal system (MyChart) by patients hospitalized from January 1, 2014 to June 30, 2017 for AF. During the study period, 11,334 unique AF admissions were identified; 50.3% were MyChart users and 49.7% were non–MyChart users. Patients who experienced inpatient mortality were excluded. The study groups were analyzed for demographic variables, comorbidities, readmission rates, and the frequency of MyChart use during the 3.5-year time frame.

**Results:** MyChart users were younger (median age, 74 years, interquartile range [IQR] 66-82 vs 77 years, IQR 68-85; *P*<0.0001) and more likely to be white (91.9% vs 84.6%; *P*<0.0001), but the sex distribution was similar between groups, with 51.8% males in the MyChart group vs 53.2% in the non–MyChart group. MyChart users had a significantly higher rate of readmission compared to non–MyChart users at 1 year (43.0% vs 32.0%, respectively; *P*<0.0001). MyChart users who were readmitted had a higher median number of logins to MyChart (121 [IQR 32-270.5]) than MyChart users who were not readmitted (91 [IQR 26-205]; *P*<0.0001). Multivariable regression analysis demonstrated that MyChart use was associated with readmission (odds ratio 1.57, 95% CI 1.49-1.70; *P*<0.0001).

**Conclusion:** Among patients with AF, MyChart use was associated with higher readmissions in this single-center cohort. Use and benefit of bespoke portals require further study.

## INTRODUCTION

Atrial fibrillation (AF), one of the most commonly encountered arrhythmias, is known to cause frequent hospitalizations, readmissions, and increased mortality among patients in the United States. The Centers for Disease Control and Prevention estimates as many as 2.7 to 6 million people nationwide suffer from AF, with the number expected to steadily increase.^1^ Additionally, >750,000 inpatient admissions are estimated to be attributable to AF with an associated financial burden of approximately $6 billion, numbers that are also expected to increase.^1,2^ Advances as of 2017 in various therapies have shown improved outcomes in AF, yet readmission rates for AF remain high.^3^ Freeman et al showed that 30-day readmission rates for AF among a large cohort of patients from 1999 to 2013 was >13%.^3^

Many health care systems offer patients access to their electronic health records and direct communication with providers through online patient portals, with the goals of improving patient care, engagement, compliance, and efficiency. Decreasing hospital readmissions by increasing patient engagement is also a reasonable expectation associated with portal use. Reminders integrated into the portal, in conjunction with cardiac rehabilitation, have shown improved outcomes and lower readmissions,^4^ but a trial of home dialysis patients showed no improvement in patient experience with online portals.^5^ While online patient portals provide a unique communication link between providers and patients, their impact on patient care—specifically resource utilization defined by readmissions—is unclear. We conducted a retrospective analysis of all AF admissions in a large tertiary care facility during a 3.5-year period and evaluated which patient characteristics, including use of the hospital's online patient portal, MyChart, were predictive of hospital readmissions.

## METHODS

The Baylor Scott and White Research Institute Institutional Review Board approved this retrospective cohort study. Informed consent of participants was not required, as this was a retrospective, minimal-risk study. All patients admitted to Baylor Scott and White Medical Center in Temple, Texas, from January 1, 2014 to June 30, 2017 were screened via *International Classification of Diseases-10* diagnosis codes for AF (148.0, 148.1, 148.2, 148.3, 148.4, 148.91, and 148.92) and for enrollment in MyChart. We included all patients whose principal or secondary diagnosis was AF, with no exclusion criteria based on patient demographics. We excluded all patients who experienced inpatient mortality. To avoid potentially missing patients who were readmitted, we defined any individual readmission stays with a primary or secondary diagnosis of AF within 1 year of previous hospitalization. A total of 11,334 patients with unique hospital stays were identified, and each stay was counted as 1 hospital admission. Diagnosis codes for diabetes mellitus, hypertension, major bleed, stroke/transient ischemic attack, congestive heart failure, and vascular disease (myocardial infarction, peripheral artery disease, and aortic atherosclerotic plaques) were abstracted, and the data were included in the patient demographic analysis. Inpatient laboratory tests were evaluated for liver disease (total bilirubin >4.0 mg/dL and aspartate transaminase >150 U/L) and renal disease (creatinine >1.4 mg/dL), and these comorbidities were also included in the analysis. Age, sex, and patient readmission data were also extracted from the electronic medical record. We matched patient MyChart data to the AF cohort and extracted the duration of online participation and logins throughout the study period.

Sample characteristics are given using descriptive statistics. Frequencies and percentages are used to describe categorical variables. Means ± SD (or medians and interquartile ranges [IQRs] where appropriate) are used to describe continuous variables. The baseline patient characteristics were contrasted in MyChart users vs non–MyChart users using paired *t* tests and multivariable logistical regression analysis to identify predictors of AF readmission.

## RESULTS

Data were collected for 11,334 patients with individual hospital admissions with either a primary or secondary diagnosis of AF during the study period. Baseline demographics are detailed in Table 1. Of the 11,334 patient admissions, the 2 cohorts had a relatively equal distribution, with 50.3% (n=5,702) MyChart users and 49.7% (n=5,632) non–MyChart users. MyChart users were younger, with a median age of 74 years [IQR 66-82] vs 77 years [IQR 68-85] for non–MyChart users (*P*<0.0001). Patients in the MyChart group were more likely to be white than non–MyChart users (91.9% vs 84.6%, respectively; *P*<0.0001) and had higher rates of hypertension (88.3% vs 86.0%, respectively; *P*=0.0002), liver disease (5.5% vs 4.2%, respectively; *P*=0.0013), and vascular disease (23.0% vs 20.0%, respectively; *P*<0.0001).

**Table 1.** Demographics, Comorbidities, and Readmissions of MyChart Users vs Non–MyChart Users (n=11,334)

Variable	MyChart Users n=5,702	Non–MyChart Users n=5,632	*P* Value
Age, years, median [IQR]	74 [66-82]	77 [68-85]	<0.0001
Male	2,954 (51.8)	2,997 (53.2)	0.13
Race, white	5,241 (91.9)	4,765 (84.6)	<0.0001
Comorbidities			
Diabetes	1,928 (33.8)	1,994 (35.4)	0.1
Hypertension	5,035 (88.3)	4,844 (86.0)	0.0002
Major bleed	985 (17.3)	840 (14.9)	0.0006
Stroke/transient ischemic attack	1,631 (28.6)	1,662 (29.5)	0.29
Congestive heart failure	2,692 (47.2)	2,715 (48.2)	0.27
Renal disease	2,418 (42.4)	2,327 (41.3)	0.23
Liver disease	314 (5.5)	237 (4.2)	0.0013
Vascular disease	1,312 (23.0)	1,127 (20.0)	<0.0001
Hospital readmission within 1 year	2,452 (43.0)	1,803 (32.0)	<0.0001

Note: Data are presented as n (%) unless otherwise indicated.

IQR, interquartile range.

Overall, 4,255 patients were readmitted to the hospital for AF within 1 year. MyChart users were readmitted at a substantially higher rate compared to non–MyChart users (43.0% vs 32.0%, respectively; *P*<0.0001).

**Figure 1.**
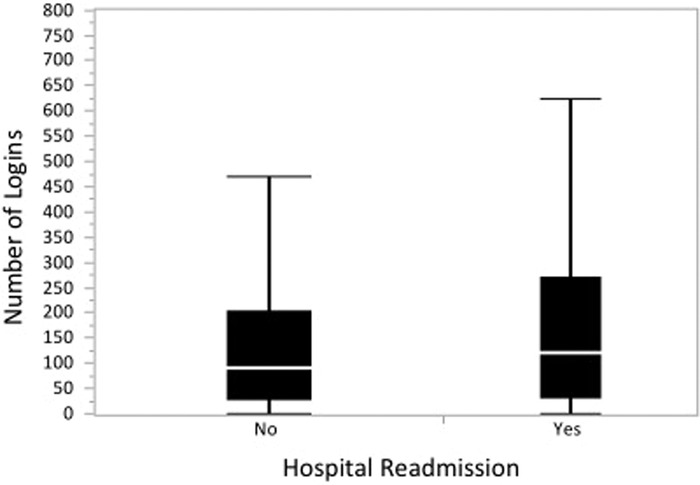
**One-way analysis of the number of logins to MyChart by hospital readmission. Readmitted patients had more frequent logins than nonreadmitted patients.**

**Figure 2.**
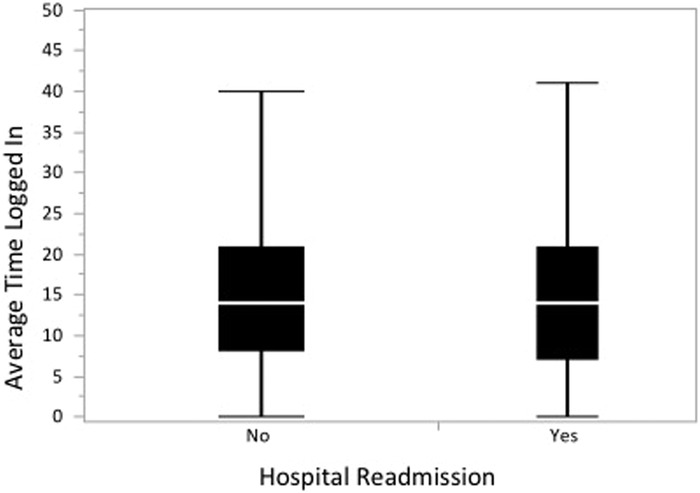
**One-way analysis of average of total minutes of time logged in by hospital readmission. Analysis of the mean time spent per login to MyChart was similar for readmitted patients and nonreadmitted patients.**

MyChart users who were readmitted had a higher median frequency of logins than MyChart users who were not readmitted (121 logins [IQR 32-270.5] vs 91 logins [IQR 26-205], respectively; *P*<0.0001) (Figure 1). MyChart users who were readmitted and MyChart users who were not readmitted had similar duration times (minutes) spent on MyChart while logged in (median 14 minutes [IQR 7-21] vs median 14 minutes [IQR 8-21]; *P=*0.43) (Figure 2). MyChart users who were readmitted had a significantly higher mean total number of logins (188.8 ± 220.0 vs 152.6 ± 191.6, respectively; *P*<0.0001) and higher mean total minutes spent logged into the patient portal (2,392.9 ± 2,955.5 vs 1,958.6 ± 2,590.6, respectively; *P*<0.0001) than MyChart users who were not readmitted. No significant difference was seen in the average time spent logged on per session between MyChart users who were readmitted and those who were not readmitted (14.6 min ± 8.7 vs 14.8 min ± 9.2, respectively; *P*=0.41). MyChart users who avoided readmission had a shorter time duration between index discharge from the hospital and initial login to the patient portal (141.4 days ± 448.1 vs 308.8 days ± 445.5, respectively; *P*<0.0001) than MyChart users who were readmitted.

Multivariable regression analysis demonstrated that MyChart use was predictive of readmission for AF (odds ratio [OR] 1.57, 95% CI 1.49-1.70; *P*<0.0001) (Table 2). Male sex (OR 1.26, 95% CI 1.17-1.38), each year of advancing age (OR 0.995, 95% CI 0.991-0.998), diabetes (OR 1.09, 95% CI 1.00-1.19), hypertension (OR 1.28, 95% CI 1.13-1.45), and congestive heart failure (OR 1.48, 95% CI 1.34-1.57) were also associated with readmission (Table 2).

**Table 2.** Multivariable Regression Analysis of Predictors of Readmission

Variable	Odds Ratio	95% CI	*P* Value
Age, per year	0.995	0.991-0.998	0.002
Male	1.26	1.17-1.38	<0.0001
Nonwhite race	1.09	0.96-7.24	0.18
Comorbidities			
Diabetes	1.09	1.00-1.19	0.04
Hypertension	1.28	1.13-1.45	0.0001
Major bleed	1.10	0.99-1.23	0.07
Stroke/transient ischemic attack	1.07	0.99-1.17	0.10
Congestive heart failure	1.48	1.34-1.57	<0.0001
Renal disease	1.04	0.96-1.13	0.49
Liver disease	1.00	0.84-1.20	0.99
Vascular disease	1.05	0.95-1.15	0.32
MyChart account	1.57	1.49-1.70	<0.0001

## DISCUSSION

Web-based patient portals are commonly believed to be an advance in patient care, but the implications of using this patient-provider link are difficult to ascertain and have yet to be fully comprehended. Our study demonstrated a significant deleterious association between patients with AF who are users of the online portal MyChart and readmissions to our hospital. We also noted more frequent logins among patients who were readmitted compared to those who avoided documented readmission, but the time spent on MyChart was similar between groups. These data are counterintuitive to common beliefs about patient portals, and the finding was unexpected.

Data regarding the efficacy of online patient portals, specifically how best to analyze their effects on patient outcomes such as rehospitalization, are scarce. Our findings were a direct contrast to a previous study by Widmer et al among patients with acute coronary syndrome who were using digital health interventions in conjunction with cardiac rehabilitation after percutaneous coronary interventions.^4^ An important note is that interventions were integrated into the portals for these patients, which is different than our AF study cohort. A benefit demonstrated in the prior Widmer et al study was increased usage of the portal when digital health interventions were incorporated into the portal,^4^ which we were unable to account for in the present study because no portal interventions were included for the AF cohort. One potential explanation for this difference in portal usage could be because the present study had no portal interventions, patients potentially only contacted their providers when they were having difficulties with AF, and those contacts could have led to more readmissions on the advice of the physicians through the portal.

Our finding of increased readmissions was similar to a 2016 study investigating portal usage among a large cohort (n=2,975) of patients discharged from the hospital with a diagnosis of acute myocardial infarction, pneumonia, or congestive heart failure.^6^ The researchers found that active users of their patient portal system had a dramatically higher rate of readmission (66%) than nonusers of the portal system. Their study cohort was hypothesized to have more medical problems and may have benefited from readmissions. The propensity to engage via the portal may be associated with individuals’ perceptions or the true severity of their disease processes and hence could potentially function as a risk marker for readmission.

Our findings raise the question of what the true consequences of online patient portal use are and how they impact patient care. For instance, are patients who use these portals more likely to have an increased need for hospitalization, or does web access to a provider facilitate more hospital readmissions? A randomized controlled trial among the veteran population aimed at decreasing readmissions found that increased exposure to primary care interventions actually increased readmissions to the hospital.^7^ The authors hypothesized that having more access to a provider for voicing complaints could have led to higher readmissions among the study cohort. Additionally, the patients in the intervention group were more satisfied with their care than those in the control group despite their increased readmission rates. An important note is that the access to primary care interventions in this trial was through direct patient care and not through web communication, which may limit the generalizability to our study.^7^ A reasonable assumption, however, is that frequent interactions between providers and their patients via online portals are likely to produce similar results and may help validate some of the data we have demonstrated.

As online portals have become more frequently used, digital health trials have noted potential benefits of usage frequency on patient outcomes. The South Asian Heart Risk Assessment (SAHARA) trial (2016) showed that digital health interventions did not lower the risk of cardiac events among a highly educated population at high risk for cardiac events based on genetic predilection.^8^ Specifically, no statistical difference was found between patients at high risk for myocardial infarction in the digital health arm and in the control arm, a finding that was not predicted based on results from the feasibility portion of the trial. A possible confounder could be the difference in the number of digital interventions or digital messages received during the feasibility trial (approximately 4 per week) compared to the number in the randomized controlled trial (2 interventions per week).^8,9^ This finding may suggest, as previously mentioned, a benefit from increased usage of the portal.^4^

When we analyzed the frequency of logins for readmitted patients vs nonreadmitted patients, we found, somewhat surprisingly, more frequent logins and a greater total amount of time spent on the portal among patients who were readmitted. These results suggest that more frequent portal usage may be an interesting surrogate to monitor for increased risks of readmission and could be an interesting method to identify patients at high risk for emergency department visits and/or readmissions. Conversely, patients who engaged with the online portal earlier after dismissal tended to avoid readmission. This finding suggests that early and meaningful engagement with online portals—not necessarily the quantity of time spent on portals—may be key to avoiding readmissions.

Viewing the frequency of logins as a surrogate for portal utilization is intuitive but may be an oversimplification. In a systematic review of 62 studies, Sieverink et al identified the difficulty of determining the intended use of digital heath technologies, and thus the concept of *adherence* to digital health is likely to be incorrectly used.^10^ Sieverink et al suggested standardization of adherence in terms of intended use and valid measures for adherence to understand the influence on patient outcomes. Being able to accurately quantify and describe digital health use and adherence could benefit the understanding of the risks and benefits associated with online patient portals and best practices.

Our study has the potential for unforeseen confounders commonly encountered in retrospective data, and as such, our data should be hypothesis generating. Also, our study possibly did not account for bias from patients who experienced out-of-hospital mortality and therefore did not use MyChart. Furthermore, we potentially may not have captured all patient readmissions if patients were readmitted to outside hospitals. Finally, our study has a risk of ascertainment bias because our cohort had the primary diagnosis of AF, and subsidiary/complicating diagnoses might have been missed.

## CONCLUSION

The results of this study demonstrate higher rates of hospital readmissions in patients with AF who were users of the online patient portal MyChart vs nonusers. Online patient portals provide patients unique access to their providers that may potentially have unintended consequences such as increased readmissions. As use of digital health technology becomes more prevalent, studies are needed to evaluate how patients and providers use patient portals and their impact on patient care. Understanding patients’ engagement with online portals is difficult to assess, and further studies are required before declaring that these portals lead to undesired outcomes such as increased readmissions.

## Notes

This article meets the Accreditation Council for Graduate Medical Education and the American Board of Medical Specialties Maintenance of Certification competencies for Patient Care and Medical Knowledge.
